# Characterization of a novel zinc transporter ZnuA acquired by *Vibrio parahaemolyticus* through horizontal gene transfer

**DOI:** 10.3389/fcimb.2013.00061

**Published:** 2013-10-10

**Authors:** Ming Liu, Meiying Yan, Lizhang Liu, Sheng Chen

**Affiliations:** ^1^Department of Applied Biology and Chemical Technology, The Hong Kong Polytechnic UniversityHong Kong, China; ^2^Food Safety and Technology Research Center, Hong Kong PolyU Shen Zhen Research InstituteShenzhen, China; ^3^State Key Laboratory for Infectious Disease Prevention and Control, National Institute for Communicable Disease Control and Prevention (ICDC), Chinese Center for Disease Control and PreventionBeijing, China

**Keywords:** *Vibrio parahaemolyticus*, horizontal gene transfer, znuA, fitness, virulence

## Abstract

*Vibrio parahaemolyticus* is a clinically important foodborne pathogen that causes acute gastroenteritis worldwide. It has been shown that horizontal gene transfer (HGT) contributes significantly to virulence development of *V. parahaemolyticus*. In this study, we identified a novel *znuA* homolog (*vpa1307*) that belongs to a novel subfamily of ZnuA, a bacterial zinc transporter. The *vpa1307* gene is located upstream of the *V. parahaemolyticus* pathogenicity island (Vp-PAIs) in both *tdh*-positive and *trh*-positive *V. parahaemolyticus* strains. Phylogenetic analysis revealed the exogenous origin of *vpa1307* with 40% of *V. parahaemolyticus* clinical isolates possessing this gene. The expression of *vpa1307* gene in *V. parahaemolyticus* clinical strain VP3218 is induced under zinc limitation condition. Gene deletion and complementation assays confirmed that *vpa1307* contributes to the growth of VP3218 under zinc depletion condition and that conserved histidine residues of Vpa1307 contribute to its activity. Importantly, *vpa1307* contributes to the cytotoxicity of VP3218 in HeLa cells and a certain degree of virulence in murine model. These results suggest that the horizontally acquired *znuA* subfamily gene, *vpa1307*, contributes to the fitness and virulence of *Vibrio* species.

## Introduction

*Vibrio parahaemolyticus* is a motile and facultative anaerobe that frequently inhabits in estuarine and marine environments. It is a well-known pathogen that leads to acute gastroenteritis worldwide, especially in areas with high level consumption of seafood such as raw oysters and shrimps (Blake et al., 1980).

The pathogenicity of this pathogen is highly associated with the thermostable direct hemolysin (TDH), TDH-related hemolysin (TRH), and two type III secretion systems (T3SSs), T3SS1 and T3SS2 (Shirai et al., 1990; Hiyoshi et al., 2010). TDH is a pore-forming toxin that leads to the lysis of human erythrocytes, activation of calcium influx, disruption of cytoskeleton and epithelial barrier, while TRH has only been linked to hemolytic activity. T3SS1 is prevalent in all *V. parahaemolyticus* strains, whereas T3SS2 is more popular among clinical isolates (Meador et al., 2007). Comparative genomic analysis of pre-pandemic and pandemic *V. parahaemolyticus* strains, as well as molecular profiling studies, revealed that the organization of mobile gene cassettes and pathogenicity islands were divergent in these *V. parahaemolyticus* strains and that the emergence of pandemic strain could be associated with the recombination events and novel gene acquisition (Hurley et al., 2006; Gonzalez-Escalona et al., 2008; Han et al., 2008; Caburlotto et al., 2011; Chen et al., 2011; Yan et al., 2011; Garcia et al., 2012; Gennari et al., 2012; Gavilan et al., 2013; Ottaviani et al., 2013; Theethakaew et al., 2013; Turner et al., 2013). The genetic divergence of *V. parahaemolyticus* strongly correlates with their diverse virulence potentials (Vongxay et al., 2008; Caburlotto et al., 2010).

T3SS2 is located within the pathogenicity island (Vp-PAI), implying the acquisition of T3SS2 via horizontal gene transfer (HGT) (Okada et al., 2009). Recent works also support that T3SS2 is not only transferable among *V. parahaemolyticus* strains but also among other *Vibrio* spp. (Dziejman et al., 2005; Caburlotto et al., 2009; Okada et al., 2010). Functional analysis of genes encoded in the T3SS2 gene cluster revealed that T3SS2 in *V. parahaemolyticus* contributes to its colonization and competition to protists in aquatic environment (Matz et al., 2011; Ritchie et al., 2012). Apart from the genes of T3SS2, other horizontally acquired genes can also contribute to the virulence of *V. parahaemolyticus*. *VpaH* in *V. parahaemolyticus* TH3996, an acquired gene through HGT, can significantly enhance its motility, biofilm formation and adherence (Park et al., 2005). This observation has prompted the need to characterize novel horizontally acquired virulence factors in *V. parahaemolyticus*.

Zinc is an important component for many bacterial metalloenzymes. Intracellular pathogens require zinc for invasion, survival, and replication in the host. However, zinc concentration in the host is very low, therefore, it is essential for pathogenic bacteria to take up zinc effectively in the host (Hantke, 2005). Most pathogenic bacteria take up zinc using single or multiple zinc transportation systems. ZnuACB is the most important high-affinity zinc acquisition system in many bacteria, where ZnuA is responsible for zinc binding, ZnuB is for transportation of zinc across the inner membrane, and ZnuC works as an ATPase providing energy for zinc intake process (Hantke, 2005). ZnuACB was shown to be essential for virulence of *Brucella abortus*, *Campylobacter jejuni*, *Moraxella catarrhalis*, *Salmonella enterica*, and *Haemophilus ducreyi* (Lewis et al., 1999; Yang et al., 2006; Ammendola et al., 2007; Davis et al., 2009; Murphy et al., 2013), but not for uropathogenic *Escherichia coli*, *Proteus mirabilis*, or *Yersinia pestis* (Sabri et al., 2009; Desrosiers et al., 2010; Nielubowicz et al., 2010). This is due to the possessing of other zinc uptake genes, such as *zupT*, encoding a low-affinity zinc acquisition protein in *E. coli* (Sabri et al., 2009). In *V. parahaemolyticus*, the mechanisms of zinc transportation and its contribution to the pathogenesis of *V. parahaemolyticus* are not well-defined.

In this study, we identified a unique *znuA* homolog (*vpa1307*) that represents a novel subfamily of ZnuA in *V. parahaemolyticus*. *Vpa1307* is localized upstream of Vp-PAIs in both *tdh*-positive *V. parahaemolyticus* RIMD2210633 and *trh*-positive *V. parahaemolyticus* TH3996 strains. Phylogenetic analysis suggested that *vpa1307* is acquired by *V. parahaemolyticus* through HGT. The role of Vpa1307 as ZnuA was confirmed and its contribution to the pathogenesis of *V. parahaemolyticus* was determined.

## Materials and methods

### Bioinformatics analysis

Multiple sequence alignments were performed by the use of Clustal W2. Three-dimensional (3D) structure was predicated and modeled using Swiss-model. Structural alignment was generated using TM-align servers from Zhang's lab at University of Michigan. Phylogenetic analysis was performed using MEGA version 5 after multiple alignment of the data via CLUSTAL_X. Distances were obtained using options according to Kimura's two-parameter model and clustering was performed by using the neighbor-joining method. The topology of the neighbor-joining phylogenetic tree was evaluated by using bootstrap resampling with 1000 replications.

### Bacterial strains, plasmid, and growth conditions

Plasmids pDM4 and pMMB207 were used for gene deletion and complementation experiments, respectively. *E. coli* SY327 λ*pir* was used for conjugation (Miller and Mekalanos, 1988). Clinical *V. parahaemolyticus* strains were obtained from hospitals in Hong Kong. Other strains and plasmids used in this study were listed in Table 1. *V. parahaemolyticus* was cultured in LB medium supplemented with 2.5% sodium chloride (NaCl) at 37°C. Chloramphenicol (25 μg/ml for *E. coli* and 5 μg/ml for *V. parahaemolyticus*), kanamycin (50 μg/ml for *E. coli*), and 1 mM Isopropyl β-D-1-Thiogalactopyranoside (IPTG) were supplied if necessary. Zinc depletion was carried out using specific zinc chelator, *N,N,N′,N′*-Tetrakis (2-pyridylmethyl) ethylenediamine (TPEN, Sigma) dissolved in ethanol.

**Table 1:** **Bacteria and plasmids used in this study**.

**Strain/plasmid**	**Description**	**References or source**
*E. coli* SY327 λ*pir*	Δ(*lac-pro*) *argE*(Am) *rif malA recA56* λ*pir*	Miller and Mekalanos, 1988
*V. parahaemolyticus* VP3218	Clinical isolate, *tdh*^+^, *t3ss1*^+^, *t3ss2*^+^	Prince Wales Hospital Hong Kong
Δ *vpa1307*	*vpa1307* gene deletion mutant	This study
Δ *vpa1307*::p*vpa1307*	Δ *vpa1307* complemented with *vpa1307*gene	This study
Δ *vpa1307*::p*vpa1307*(H^69^A H^148^A)	Δ *vpa1307* complemented with H69 H148 mutated *vpa1307*gene	This study
**PLASMIDS**		
pDM4	Cm^r^; suicide vector with an R6K origin and *sacBR* genes from *Bacillus subtilis*	Zhou et al., 2010
pMMB207	RSF1010 derivative, *IncQ lacI*^q^ Cm^r^ P*tac oriT*	Zhou et al., 2010
pPK2013	Km^r^ Tra^+^ Mob^+^, ColE1 replicon	Liverman et al., 2007

### Construction of deletion and complementary strains

The *vpa1307* gene was deleted from *V. parahaemolyticus* strain VP3218 by homologous recombination using the methods described previously (Liverman et al., 2007; Zhou et al., 2010). Briefly, the upstream and downstream sequences of *vpa1307* gene were amplified using primers vpa1307-1F/vpa1307-1R and vpa1307-2F/vpa1307-2R, respectively (Table 2). These two fragments were used as templates for the second round of PCR using primers vpa1307-1F/vpa1307-2R. The purified overlapping PCR product was digested and cloned into the same digested suicide vector, pDM4. *E. coli* SY327 λ *pir* carrying the recombinant plasmid, the helper plasmid pPK2013, and *V. parahaemolyticus* strain VP3218 were mixed (5:5:1, v/v/v), spun down and resuspended in 100 μ l LB broth, poured onto a filter on LB agar plate, and incubated overnight. The bacteria on the filter were resuspended, spread on Thiosulfate-citrate-bile salts-sucrose agar (TCBS) containing 5 μg/ml chloramphenicol to select transconjugants. Randomly selected transconjugants were cultured on LB agar in the presence of 5% sucrose and subjected to repeated serial passages. The knockout mutant, Δ *vpa1307* was obtained.

**Table 2:** **Primers used in this study**.

**Primer name**	**Sequence or reference**
vpa1307-1F	CCGCTCGAGGAGGGTTCTGACGTTGGTGT
vpa1307-1R	GTGTATTCTGTCATGATCAATTAGAACGCATGAGCACCGT
vpa1307-2F	ACGGTGCTCATGCGTTCTAATTGATCATGACAGAATACAC
vpa1307-2R	CGAGCTCACGCAAAAAGCACCATTACC
vpa1307com-F	CGAGCTCTAAGGAGGTAGGATAATATTGGGGCGCACGGTGCTC
vpa1307com-R	CGGGATCCTCAAAACTTCACAGCGCT
vpa1307-F	TTGGGGCGCACGGTGCTCAT
vpa1307-R	TCAAAACTTCACAGCGCT
rtvpa1307-F	TACGCTGCCAGTTTTGTACG
rtvpa1307-R	GATCCGCAACTTGAACCATT
rt16S-F	GGAAGGTAGTGTAGTTAATAGC
rt16S-R	GATGTCAAGAGTAGGTAAGGT
H69A-F	CCGATAAACAAGATCCAGCTTACGTGCAAGCTCGCC
H69A-R	GGCGAGCTTGCACGTAAGCTGGATCTTGTTTATCGG
H148A-F	GCGCATGGTAATCCGGCCGTGCAGTTTGCGG
H148A-R	CCGCAAACTGCACGGCCGGATTACCATGCGC

To construct the complementary strain, the *vpa1307* gene with additional ribosome-binding site was amplified using primers vpa1307com-F and vpa1307com-R (Zhou et al., 2010) (Table 2). PCR product was digested and cloned into the same digested pMMB207 to create pMMB207:*vpa1307*. This recombinant plasmid was transformed into *E. coli* SY327 λ *pir* and then conjugated into Δ*vpa1307* with the presence of helper plasmid pPK2013 carrying *E. coli* SY327 λ *pir*. Transconjugants were selected on TCBS containing 5 μg/ml chloramphenicol and the strain Δ *vpa1307*::p*vpa1307* was obtained.

Site-directed mutagenesis was generated using GENEART® Site-Directed Mutagenesis kit (Invitrogen Co., NY, USA) with primer pairs H69A-F/H69A-R and H148A-F/H148A-R, (Table 2). Plasmid pMMB207:*vpa1307* was used as template. Successful mutations were confirmed by sequencing. Δ *vpa1307*::p*vpa1307 (*H^69^A, H^148^A) was obtained by the use of the method described above.

### RT-PCR, PCR, and growth assay

Thirty-five micrometers TPEN was added to wild type (WT) log-phase *V. parahaemolyticus* culture. After induction for 30 min, 1 ml culture was collected and used to extract RNA using Trizol (Invitrogen) following the manufacturer's instructions. DNA was removed from the sample with DNase (Turbo DNase, Ambion) according to the manufacturer's instructions. 0.5 μg RNA was used as template using Superscript one-step RT-PCR system (Invitrogen). No TPEN culture was used as negative control. Primers rtvpa1307-F/rtvpa1307-R and rt16S-F/rt16S-R were used, respectively (Table 2). Primers vpa1307-F and vpa1307-R (Table 2) were used for screening the distribution of *vpa1307* in *V. parahaemolyticus* clinical isolates by PCR approach. The *tdh* and *trh* genes were also screened by PCR.

For growth assay, overnight *V. parahaemolyticus* culture was diluted in LB broth and grown to the exponential growth phase (OD_600_ ≈ 0.6–0.7). The cells were diluted 1:100 into fresh LB broth with or without 35 μ M TPEN, respectively and grown at 37°C with shaking (250 rpm). OD600 was monitored at specific time points. A similar procedure was used in relative growth assay, except that OD_600_ was only monitored at 6 h. 1 mM IPTG plus 5μg/ml chloramphenicol was added when culturing the complementary strains. Relative growth rate was calculated as culture grown with TPEN to that grown without 35 μ M TPEN.

### Cytotoxicity assay

HeLa cells were washed five times with PBS to completely wash the serum-off before bacteria was added and incubated in DMEM (without serum and antibiotics). Overnight *V. parahaemolyticus* strains were diluted 100-fold using fresh LB broth and grown at 37°C for 4 h. Cultures were then collected, washed, resuspended in DMEM (without serum) and used to infect HeLa cells at a multiplicity of infection (MOI) of ~50 cfu per cell. Supernatants were collected at specific time points and the amounts of LDH released were determined using CytoTox 96 Non-Radioactive Cytotoxicity kit (Promega) following the manufacturer's instructions. Percentage of cytotoxicity was calculated using formula: (test LDH release—spontaneous release)/maximal release. Test LDH release represents the LDH release after infection with different *V. parahaemolyticus* strains; spontaneous release represents the baseline cell LDH release without infecting with any bacteria, whereas maximal release represents the release of LDH when cells were lysed using lysis solution from the kit.

### Murine infection assay

*V. parahaemolyticus* strains (10^8^ CFU) were intraperitoneally injected into 6- to 10-week-old C57BL/6 mice (*n* = 10) as previously described (Hiyoshi et al., 2010; Pineyro et al., 2010; Whitaker et al., 2012) and mice that were alive were measured at the indicated time points. Three independent replicated experiments were performed. The animal experiments were conducted in the National Institute for Communicable Disease Control and Prevention, Chinese Center for Disease Control and Prevention (CDC) following the guidelines and policies approved by the Chinese CDC.

## Results

### Bioinformatics analysis of the *vpa1307* gene and its distribution in *V. parahaemolyticus*

**Figure 1:**
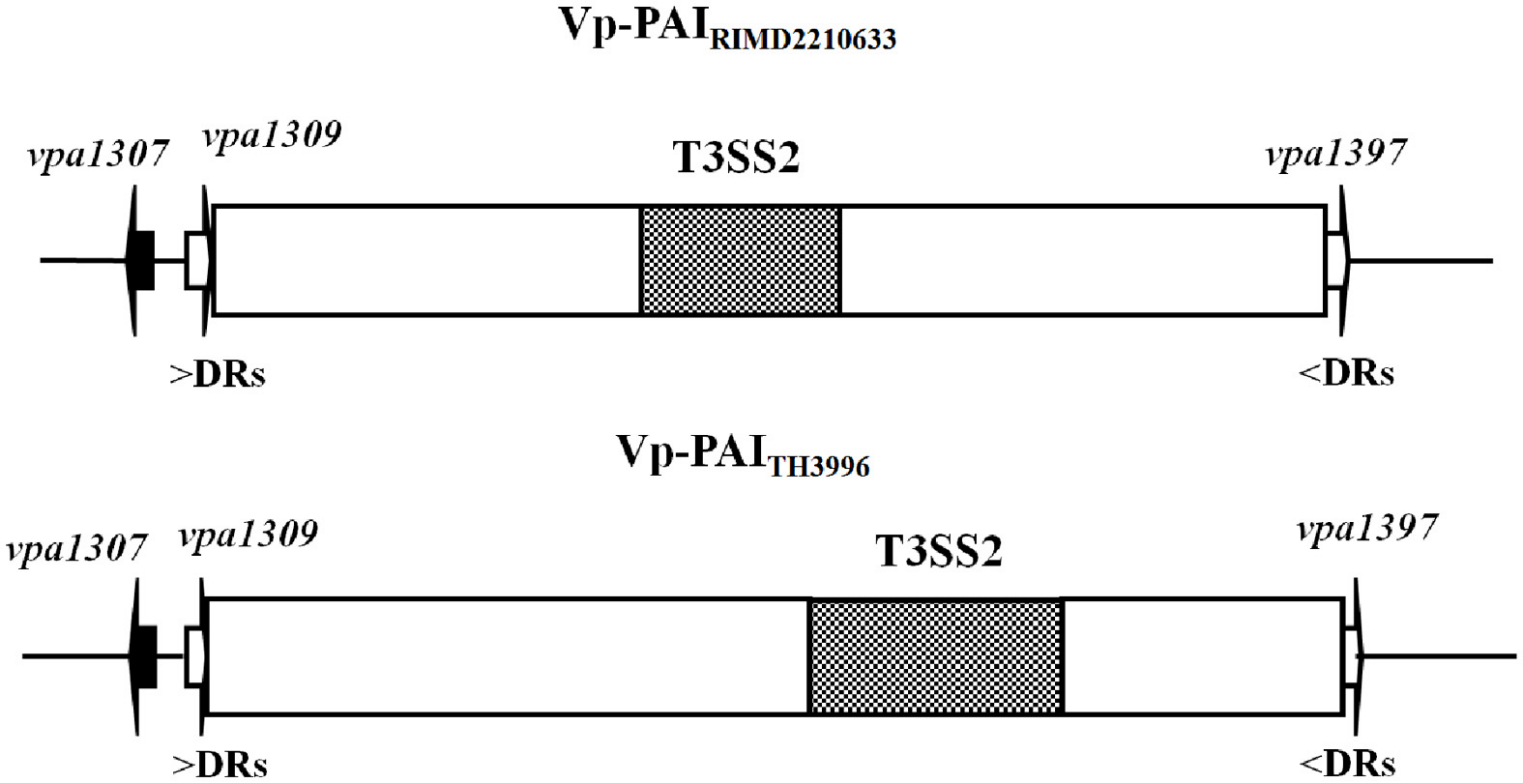
**Schematic of *vpa1307* gene location in *V. parahaemolyticus* RIMD2210633 and TH3996.** Vp-PAI (open box) is flanked by direct repeats (DRs, 5′-AACTC-3′). The white arrows (*vpa1309* and *vpa1397*) indicate the first and last genes outside the VP-PAI. The *vpa1307* gene and T3SS2 gene cluster are also indicated.

Since PAI is important for the virulence, we focused on genes related to Vp-PAI (Dobrindt et al., 2004). After a close examination of the Vp-PAI region from *tdh*-positive *V. parahaemolyticus* RIMD2210633, we found a hypothetical gene, *vpa1307* that is localized upstream of the Vp-PAI. A similar *vpa1307* was also identified upstream of Vp-PAI of a *trh*-positive *V. parahaemolyticus* TH3996 (Figure 1).

**Figure 2:**
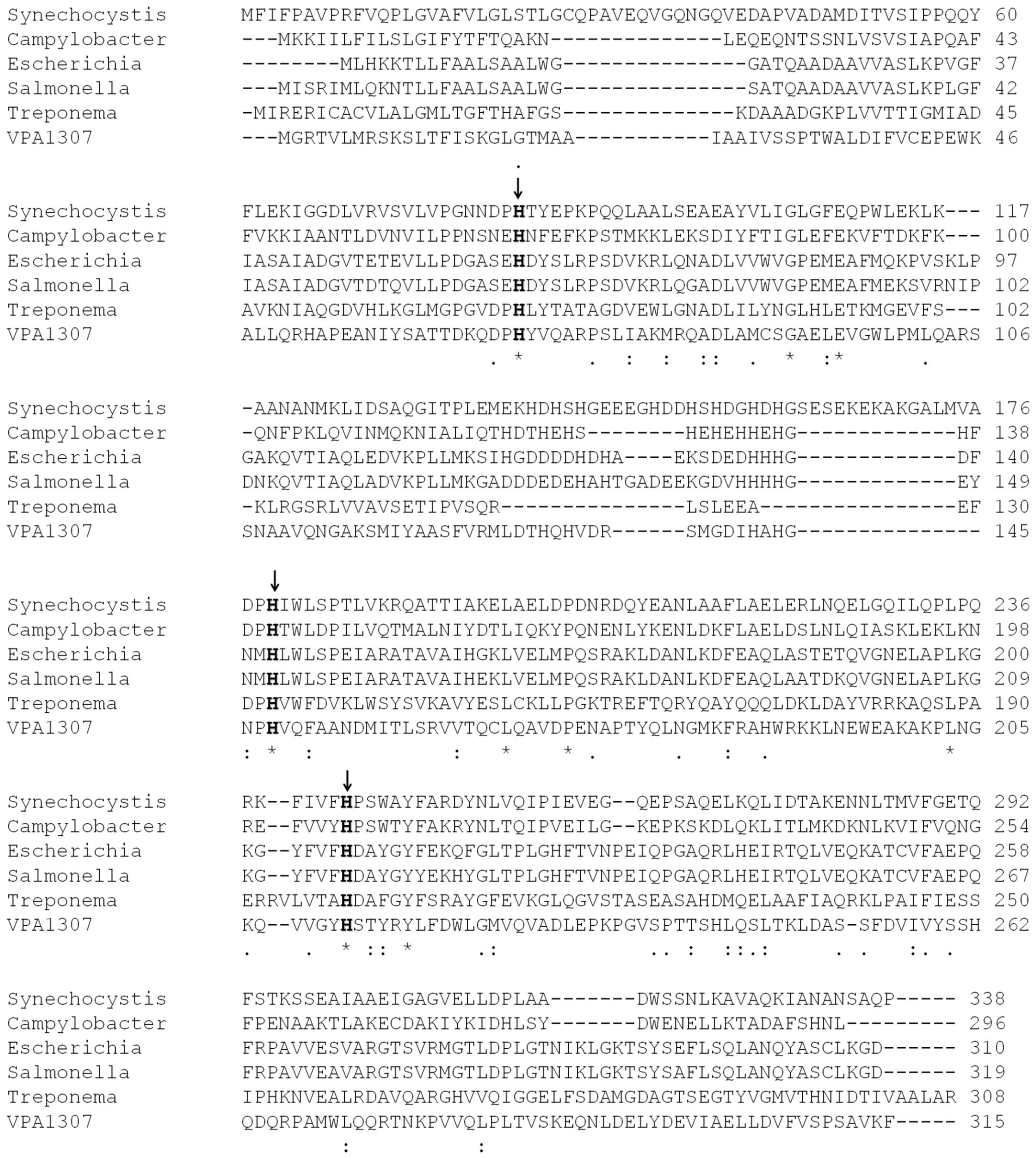
**Multiple sequence alignment of Vpa1307 and ZnuA proteins.** The amino acid sequences (GenBank accession No.CAB72627, P73085, AAC74927, Q8Z5W7, AAC45725, and Vpa1307 from *C. jejuni*, *Synechocystis* sp., *E. coli*, *S. enterica*, *Treponema pallidum*, and *V. parahaemolyticus*, respectively) were aligned using the CLUSTAL W2. Three conserved histidine residues were indicated by black arrows.

**Figure 3:**
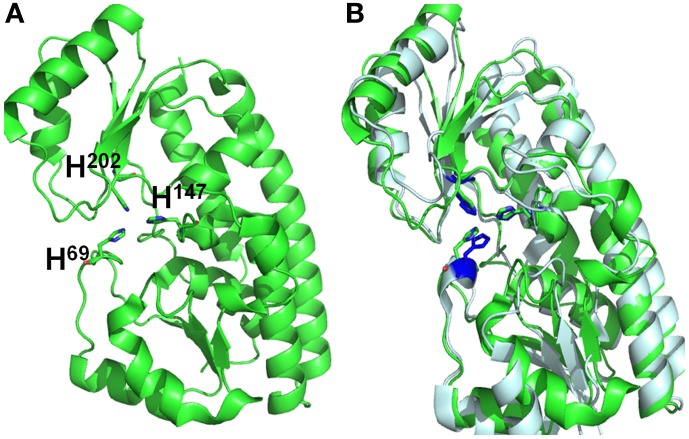
**Structural alignments between VPA1307 and ZnuA from *Synechocystis* sp. (A) VPA1307 modeled structure.** Structure of VPA1307 was modeled using SWISS-MODEL program and three conserved histidine residues were labeled. **(B)** Structural comparison of VPA1307 (green) and the crystal structure of ZnuA from *Synechocystis* sp. (PDB accession number 1PQ4) template (gray). A TM score of 0.97 was obtained over 252 aligned residues.

BLAST analysis showed that Vpa1307 shares 23% amino acid sequence identity to the zinc binding protein from *V. cholerae* O1 biovar EI Tor strain N16961. In addition, Vpa1307 possesses three conserved histidine residues, H^69^, H^148^, and H^202^ that are the hallmark of ZnuA family of proteins (Figure 2). It was shown that residues H^69^, H^148^, and H^202^ are critical for zinc binding and activity (Banerjee et al., 2003; Li and Jogl, 2007; Loisel et al., 2008; Yatsunyk et al., 2008; Ilari et al., 2011). The 3D structure of Vpa1307 was modeled and aligned with the crystal structure of ZnuA from *Synechocystis* sp. PCC 6803, even through the similarity between these two proteins is only 24%. The TM score, an algorithm to calculate the structural similarity of two protein models, is 0.97, which strongly suggests that Vpa1307 is likely to be a member of ZnuA family (Figure 3).

**Figure 4:**
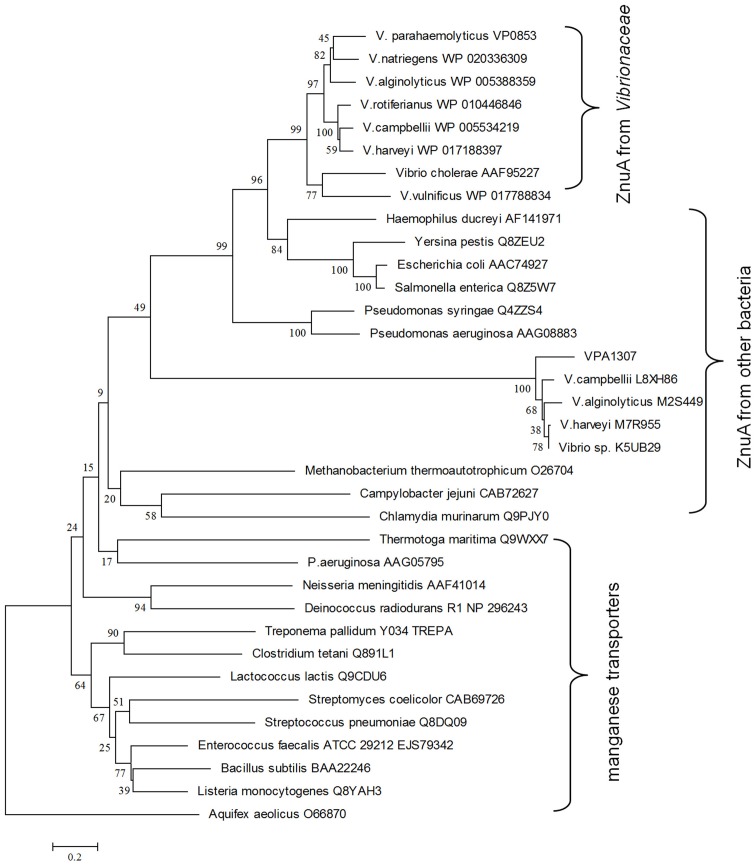
**Neighbor-joining tree of VPA1307 and related genes.** The protein sequences were obtained from NCBI except that L8XH86, M2S449, M7R955, and K5UB29 were obtained from EBI. ZnuA homologs were represented in red (except that of *Aquifex aeolicus*) and manganese transporters were represented in black. Bootstrap values (>50%) are shown at branch nodes. ZnuA homolog protein sequence from *Aquifex aeolicus* was used as outgroup. Bar, 0.2 difference at the amino acid level.

To explore the evolution history of Vpa1307, a phylogenetic tree was constructed (Figure 4). The neighbor-joining phylogenetic tree showed that Vpa1307 together with its four homologs from other *Vibrio* spp. fell within the lineage of ZnuA family and formed a distinct cluster within members of ZnuA from other genera. Intriguingly, the phylogenetic analysis also showed that Vpa1307 was excluded from the *Vibrionaceae* clade of ZnuA, suggesting an exogenous origin of Vpa1307 and representing a novel subfamily of ZnuA. The data suggested that *vpa1307* is very likely acquired by *V. parahaemolyticus* through HGT.

### Prevalence of *vpa1307* in *V. parahaemolyticus* clinical isolates

Given that *vpa1307* group genes were exogenously acquired by *Vibrio* spp., the prevalence of this gene among *V. parahaemolyticus* strains was evaluated. Our data showed that the *vpa1307* gene was detectable in 8 out of 20 (40%) of the *tdh*-positive strains but not in *tdh*- and *trh*-negative strains, suggesting the exogenous origin of *vpa1307*.

### Role of Vpa1307 on *V. parahaemolyticus* growth

**Figure 5:**
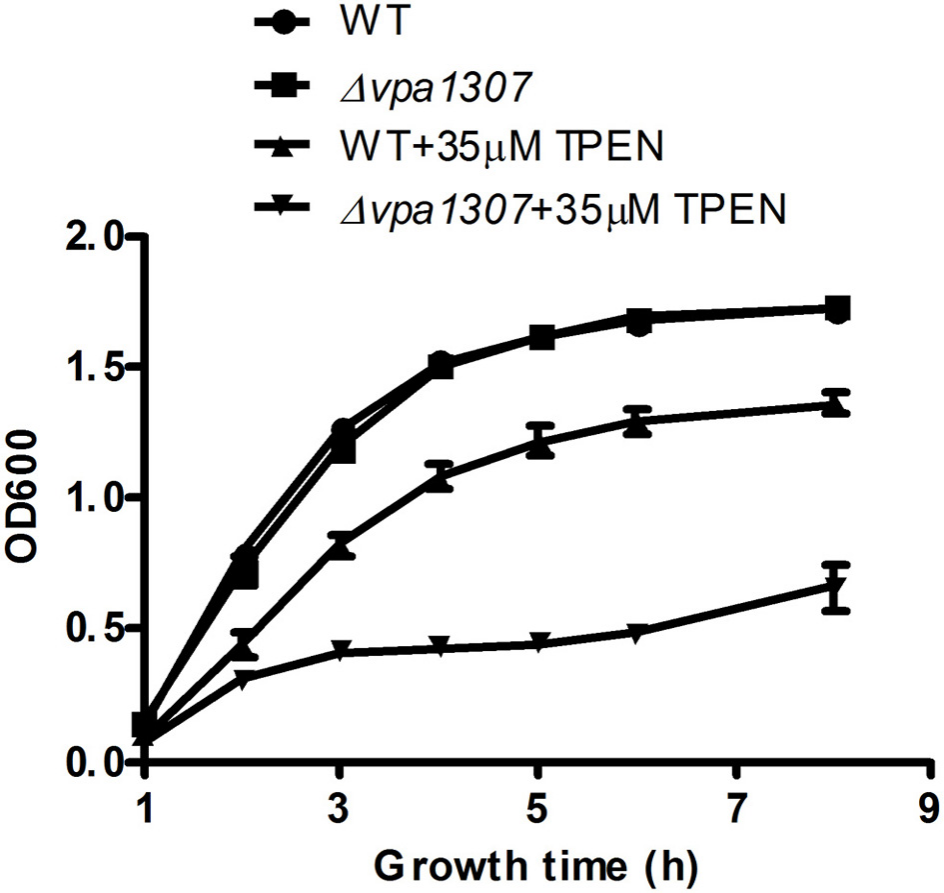
**Growth rates of *V. parahaemolyticus* strains.** *V. parahaemolyticus* strains were cultured in LB or LB supplemented with 35 μ M TPEN. *V. parahaemolyticus* growth (OD600) was monitored. The data represents three independent experiments ± the SD.

**Figure 6:**
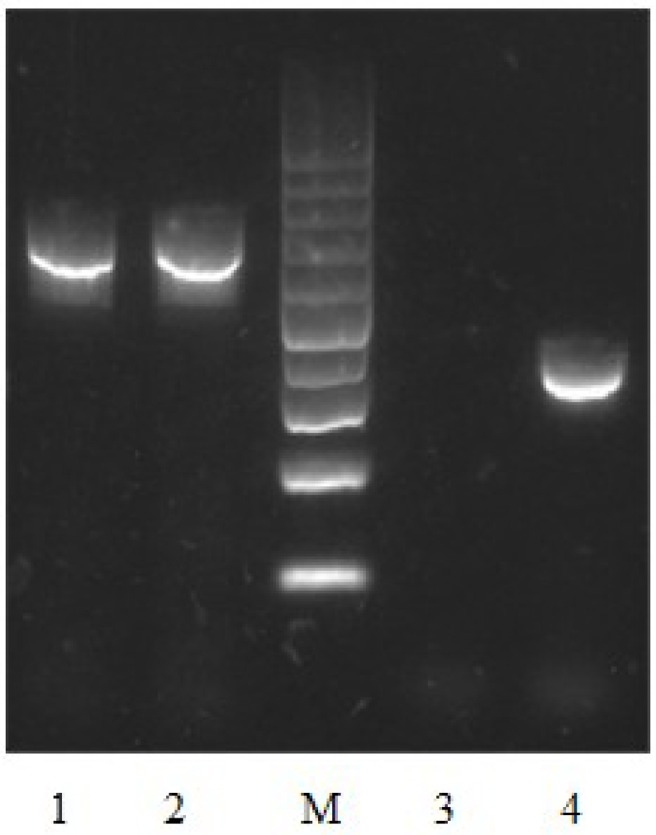
**Detection of *vpa1307* transcript by RT-PCR in *V. parahaemolyticus.*** *V. parahaemolyticus* was grown in LB and RNA was extracted after incubation with/without 35 μ M TPEN. 16S RNA was used as a loading control. Lane 1, 16S RNA (with TPEN); lane 2, 16S RNA (without TPEN); lane 3, *vpa1307* (without TPEN); lane 4, *vpa1307* (with TPEN); lane M, 100-bp maker (Thermo Scientific).

To test the contribution of Vpa1307 to *V. parahaemolyticus* growth, both WT *V. parahaemolyticus* VP3218 clinical strains and the *vpa1307* deletion mutant, Δ *vpa1307*, were grown in normal and zinc depletion conditions. All test strains showed similar growth rate in normal medium, while the growth of Δ*vpa1307* was inhibited by ~70% in the medium containing 35 μ M TPEN, a zinc chelating agent, compared to growth in normal medium. However, the growth of WT was only slightly inhibited in the medium containing 35 μ M TPEN (Figure 5). This indicated that *vpa1307* contributes to the growth of *V. parahaemolyticus* under the zinc limitation condition. The data prompt us to examine the expression status of *vpa1307* in *V. parahaemolyticus*. It was shown that *vpa1307* only expressed under zinc depletion condition (35 μ M TPEN added) (Figure 6), but not in normal medium. The expression regulation feature of *vpa1307* was consistent with the contribution of *vpa1307* to *V. parahaemolyticus* growth under zinc depletion condition.

### Conserved histidine residues of Vpa1307 contribute to its activity

**Figure 7:**
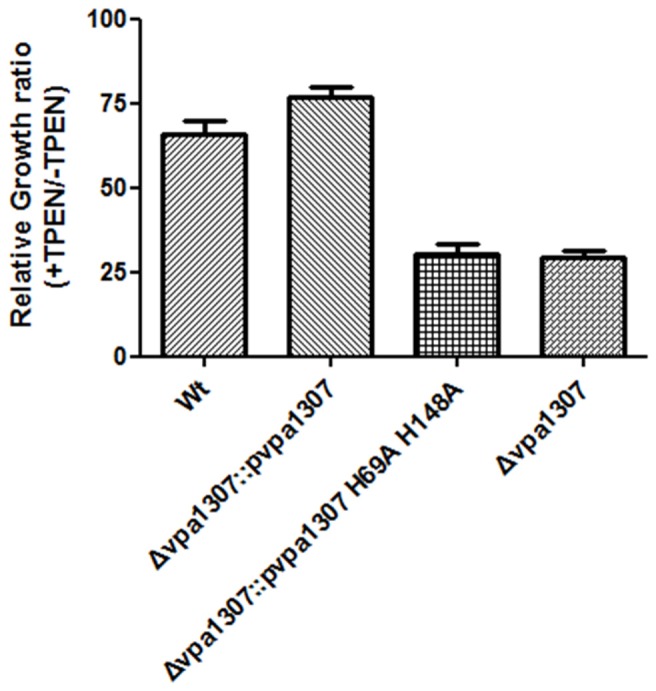
**Relative growth rate of different *V. parahaemolyticus* strains under normal and zinc depletion conditions.** Different *V. parahaemolyticus* strains as indicated were cultured in LB or LB supplemented with 35 μ M TPEN. Their growth (OD600) was monitored at 6 h and relative growth rates were calculated as culture grown with TPEN to that of grown without TPEN. The data represents three independent experiments ± the SD.

To further confirm that Vpa1307 is a homolog of ZnuA, the contribution of three conserved histidine residues to the activity of Vpa1307 was tested. An elegant design helps us to achieve this goal. First, a complementary construct was made by incorporating a *vpa1307* into plasmid pMMB207 and designated as p*vpa1307*. Second, two histidine residues, H^69^ and H^148^ that may be essential for Vpa1307 function, were mutated to alanine to obtain p*vpa1307* (H^69^A, H^148^A). Mutations of two of the three histidines are enough to inactivate a ZnuA function (Li and Jogl, 2007; Loisel et al., 2008; Ilari et al., 2011). These two constructs were then used to complement the loss of function by *vpa1307* deletion mutant, Δ*vpa1307.* As showed in Figure 7, compared to WT *V. parahaemolyticus* VP3218, *vpa1307* deletion mutant, Δ *vpa1307*, showed about 25% of growth rate. When complemented with *vap1307*, Δ *vpa1307*::p*vpa1307*, the growth rate of *V. parahaemolyticus* increased to ~75% compared to the WT strain. However, when complemented with the *vpa1307* double histidine mutant Δ *vpa1307*::p*vpa1307* (H^69^A, H^148^A), the growth rate of *V. parahaemolyticus* remained at the same level as Δ *vpa1307* (25%) suggesting that the mutations, H^69^A, H^148^A, completely abolished the activity of Vpa1307. Furthermore, RT-PCR assay has confirmed that the expression level of *vpa1307* in WT *V. parahemolyticus* VP3218 strain, Δ *vpa1307*::p*vpa1307*, and Δ *vpa1307*::p*vpa1307* (H^69^A, H^148^A) were similar suggesting that the loss of function of *vpa1307* (H^69^A, H^148^A) was due to the mutation of conserved histidine residues (data not shown). These data further confirmed that Vpa1307 is a member of ZnuA.

### Vpa1307 contributes to cytotoxicity of *V. parahaemolyticus* in hela cells

**Figure 8:**
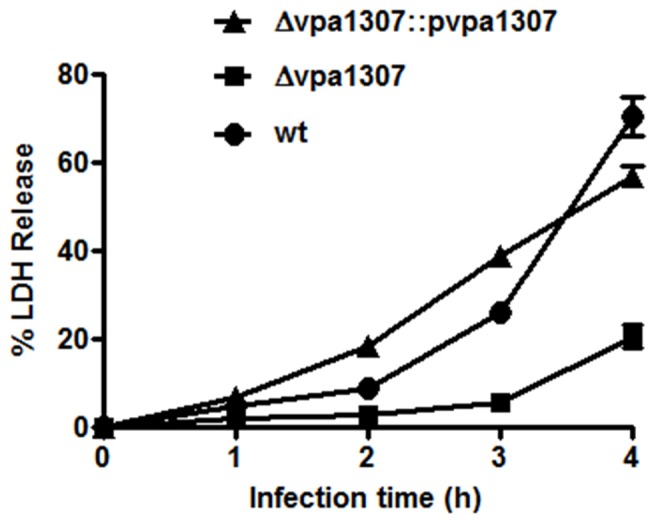
**Cytotoxic effect of vpa1037 on HeLa cells.** HeLa cells were infected with *V. parahaemolyticus* at MOI of ~50 cfu per cell. Supernatants were collected at specific time points and the amounts of LDH released were determined using CytoTox 96 Non-Radioactive Cytotoxicity kit (Promega) following the manufacturer's instructions. Percentage of cytotoxicity was calculated using formula: (test LDH release—spontaneous release)/maximal release. Test LDH release represents the LDH release after infection with different *V. parahaemolyticus* strains; *s*pontaneous release represents the baseline cell LDH release without infecting with any bacteria, whereas maximal release represents the release of LDH when cells were lysed using lysis solution from the kit. The data represents three independent experiments ± the sem.

Since ZnuA contributed to host cell infection in *B. abortus*, *M. catarrhalis*, and *S. enterica* (Yang et al., 2006; Ammendola et al., 2007; Murphy et al., 2013), we further tested whether *vpa1307* gene contributed to the virulence of *V. parahaemolyticus*. HeLa cells that were maintained in serum-free DMEM were infected with Δ *vpa1307*, Δ *vpa1307*::p*vpa1307* and WT strains, respectively. Similar to no infection cell control, strain Δ *vpa1307* did not cause notable cell rounding and detachment, while strain Δ *vpa1307*::p*vpa1307* showed more cell rounding than infected with WT strain of *V. parahaemolyticus* and all cells were detached after longer incubation (Data not shown). The cytotoxicity of *V. parahaemolyticus* was also determined by measuring the amount of LDH released from damaged cells. WT *V. parahaemolyticus* caused about 70% of LDH release, whereas Δ *vpa1307* strain caused ~20% of LDH release after 4 h infection (Figure 8). The complementation of *vpa1307*, Δ *vpa1307*::p*vpa1307*, regained its toxicity back to 60% of LDH release, a level of toxicity similar to that of WT strain. These data indicated that Vpa1307 contributes to the cytotoxicity of *V. parahaemolyticus* strain VP3218 in HeLa cells. It also suggested that acquisition of zinc from cells is required for the successful infection of *V. parahaemolyticus*.

### Vpa1307 contributes to the pathogenesis of *V. parahaemolyticus*

**Figure 9:**
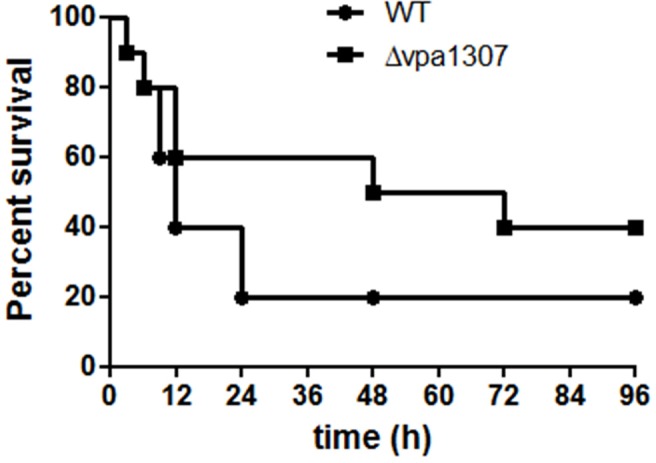
**Survival rates of murine model infected with different *V. parahaemolyticus* strains.** C57BL/6 mice (*n* = 10) were infected intraperitoneally with WT or Δ *vpa1307* strains (10^8^ CFU) and mice were monitored for the duration of 96 h. The mortality rate was measured at different time points (0, 3, 6, 9, 12, 24, 48, 72, and 96 h) for three independent experiments. Each data point in the figure represents the average of the data from three experiments. Kaplan–Meier and log rank tests were used to analyze the data.

To further evaluate the role of Vpa1307 in the pathogenesis of *V. parahaemolyticus*, mouse infection model was employed. As shown in Figure 9, during the early infection period (within 6h), both WT *V. parahaemolyticus* and *vpa1307* deletion mutant, Δ *vpa1307*, showed similar effect on mice and caused about 10% death. However, the mortality of Δ *vpa1307* was dramatically delayed and attenuated when compared with WT strain at the later infection period. At post 24 h of infection, mortality rate of WT stain reached 80%, while that of Δ *vpa1307* was about 30~50%. This indicated that the Vpa1307 contributes to the pathogenesis of *V. parahaemolyticus* at certain extent.

## Discussion

A pathogenic bacterial species is usually a group of diverse strains that inhabit in different environments. These strains usually display different infection abilities, which are highly correlated with their variation in the genomes. The evolutionary forces for the genomic flexibility involve gene gain, gene loss, gene duplication as well as mutations. It is known that HGT greatly affects the virulence of bacterium. Comparative genomic analysis of pre-pandemic and pandemic *V. parahaemolyticus* strains as well as molecular profiling studies revealed that the organization of mobile gene cassettes and PAIs were divergent in *V. parahaemolyticus* strains. The genetic divergence of this bacterium suggests that it evolves quickly in response to different pressures in the aquatic environment (Han et al., 2008; Chen et al., 2011; Gavilan et al., 2013), which results in a diverse virulence potential(Caburlotto et al., 2010). The genomic flexibility greatly affects the fitness to the hosts and the virulence potential of the pathogen. In this study, we identified and characterized a novel *znuA* homolog gene, *vpa1307* in *V. parahaemolyticus*. *Vpa1307* is localized upstream of Vp-PAI, and was annotated as an adhesion protein in strain RIMD2210633 (Makino et al., 2003). Our study revealed that *vpa1307* is a zinc transporter from a novel group of *ZnuA* family. Most interestingly, *vpa1307* was acquired by *V. parahaemolyticus* through HGT.

The exogenous origin of *vpa1307* was confirmed by studying the prevalence of this gene in clinical strains; our results show that this gene is uniquely present in *tdh*-positive strains but not in *tdh*- and *trh*-negative strains. Given that *tdh*-positive *V. parahaemolyticus* strains are commonly associated with clinical infections, the close association of *vpa1307* to *tdh*-positive strains may suggest that *vpa1307* could be one of the virulence factors contributing to clinical infection. Similar to what was found for the *znuA* family genes; the expression of *vpa1307* was induced in zinc limitation condition and contributed to *V. parahaemolyticus* growth under zinc starvation condition. Considering that zinc concentration is low in seawater (Bruland, 1989), the acquisition of *vpa1307* gene may facilitate *V. parahaemolyticus* to persist in the marine environment.

It has been shown that when more than one zinc uptake systems exist in the pathogenic bacterium, such as in uropathogenic *E. coli*, *P. mirabilis*, *Y. pestis*, and *Listeria monocytogenes*, deletion of one of them did not affect their virulence *in vivo* (Sabri et al., 2009; Desrosiers et al., 2010; Nielubowicz et al., 2010; Corbett et al., 2012). Instead, the additional zinc acquiring systems contributed to the competitive advantage, such as in uropathogenic *E. coli* and *P. mirabilis* (Sabri et al., 2009; Nielubowicz et al., 2010). Given that many *V. parahaemolyticus* strains harbored more than one *znuA* homolog genes; it is not surprising to see that Vpa1307 only contributed partially to the pathogenesis of *V. parahaemolyticus*. It is interesting to see that Vpa1307 contributes to the cytotoxicity in HeLa cells and certain degree of pathogenesis in mice.

This is the first report of a functional exogenous *znuA* homolog acquired by *V. parahaemolyticus* via HGT. The gain of this gene might enhance the survival of *Vibrio* spp. in adverse condition. HGT has been shown to contribute to bacterial fitness in new environment and virulence of a pathogen. The reason that *V. parahaemolyticus* strains caused variable cytotoxicity is probably due to acquisition of novel virulence genes, such as *vpa1307*. Considering that chitin is abundant in the aquatic environments and that it has been shown to stimulate the process of natural competence and transformation (Meibom et al., 2005), it could be a great concern that *V. parahaemolyticus* may easily acquire other genes that can strengthen its pathogenicity or antibiotic resistance in the aquatic environments.

## Funding

This work was supported by the Chinese National Key Basic Research and Development (973) Program (2013CB127200) and the Research Fund for the Control of Infectious Diseases from the Food and Health Bureau, the Government of Hong Kong SAR (K-ZJG2 to Sheng Chen).

### Conflict of interest statement

The authors declare that the research was conducted in the absence of any commercial or financial relationships that could be construed as a potential conflict of interest.
